# Automated free speech analysis reveals distinct markers of Alzheimer’s and frontotemporal dementia

**DOI:** 10.1371/journal.pone.0304272

**Published:** 2024-06-06

**Authors:** Pamela Lopes da Cunha, Fabián Ruiz, Franco Ferrante, Lucas Federico Sterpin, Agustín Ibáñez, Andrea Slachevsky, Diana Matallana, Ángela Martínez, Eugenia Hesse, Adolfo M. García

**Affiliations:** 1 Cognitive Neuroscience Center, Universidad de San Andrés, Victoria, Buenos Aires, Argentina; 2 Consejo Nacional de Investigaciones Científicas y Técnicas (CONICET), Ciudad Autónoma de Buenos Aires, Buenos Aires, Argentina; 3 Facultad de Ingeniería, Universidad de Buenos Aires (FIUBA), Ciudad Autónoma de Buenos Aires, Buenos Aires, Argentina; 4 Latin American Brain Health (BrainLat) Institute, Universidad Adolfo Ibáñez, Santiago, Peñalolén, Región Metropolitana, Chile; 5 Global Brain Health Institute, University of California San Francisco, San Francisco, California, United States of America; 6 Trinity College Dublin, Dublin, Ireland; 7 Faculty of Medicine, Neuroscience and East Neuroscience Departments, Neuropsychology and Clinical Neuroscience Laboratory (LANNEC), Physiopathology Program – Institute of Biomedical Sciences (ICBM), University of Chile, Santiago, Chile; 8 Geroscience Center for Brain Health and Metabolism (GERO), Providencia, Santiago, Chile; 9 Hospital del Salvador and Faculty of Medicine, Memory and Neuropsychiatric Center (CMYN), Neurology Department, University of Chile, Providencia, Santiago, Chile; 10 Departamento de Medicina, Servicio de Neurología, Clínica Alemana-Universidad del Desarrollo, Las Condes, Región Metropolitana, Chile; 11 Facultad de Medicina, Departamento de Psiquiatría (Programa PhD Neurociencias), Instituto de Envejecimiento, Pontificia Universidad Javeriana, Bogotá, Colombia; 12 Centro de Memoria y Cognición, Intellectus, Hospital Universitario San Ignacio Bogotá, San Ignacio, Colombia; 13 Departamento de Salud Mental, Hospital Universitario Santa Fe de Bogotá, Bogotá, Colombia; 14 Escuela de Medicina y Ciencias de la Salud, Universidad del Rosario, Bogotá, Colombia; 15 Departamento de Matemática, Universidad de San Andres, Victoria, Buenos Aires, Argentina; 16 Facultad de Humanidades, Departamento de Lingüística y Literatura, Universidad de Santiago de Chile, Estación Central, Santiago, Chile; University of Padova: Universita degli Studi di Padova, ITALY

## Abstract

Dementia can disrupt how people experience and describe events as well as their own role in them. Alzheimer’s disease (AD) compromises the processing of entities expressed by nouns, while behavioral variant frontotemporal dementia (bvFTD) entails a depersonalized perspective with increased third-person references. Yet, no study has examined whether these patterns can be captured in connected speech via natural language processing tools. To tackle such gaps, we asked 96 participants (32 AD patients, 32 bvFTD patients, 32 healthy controls) to narrate a typical day of their lives and calculated the proportion of nouns, verbs, and first- or third-person markers (via part-of-speech and morphological tagging). We also extracted objective properties (frequency, phonological neighborhood, length, semantic variability) from each content word. In our main study (with 21 AD patients, 21 bvFTD patients, and 21 healthy controls), we used inferential statistics and machine learning for group-level and subject-level discrimination. The above linguistic features were correlated with patients’ scores in tests of general cognitive status and executive functions. We found that, compared with HCs, (i) AD (but not bvFTD) patients produced significantly fewer nouns, (ii) bvFTD (but not AD) patients used significantly more third-person markers, and (iii) both patient groups produced more frequent words. Machine learning analyses showed that these features identified individuals with AD and bvFTD (AUC = 0.71). A generalizability test, with a model trained on the entire main study sample and tested on hold-out samples (11 AD patients, 11 bvFTD patients, 11 healthy controls), showed even better performance, with AUCs of 0.76 and 0.83 for AD and bvFTD, respectively. No linguistic feature was significantly correlated with cognitive test scores in either patient group. These results suggest that specific cognitive traits of each disorder can be captured automatically in connected speech, favoring interpretability for enhanced syndrome characterization, diagnosis, and monitoring.

## Introduction

Affecting nearly 55 million people worldwide, Alzheimer’s disease (AD) and behavioral variant frontotemporal dementia (bvFTD) are the most prevalent forms of dementia [1,2]. These syndromes differ in neurological and clinical aspects. AD typically entails temporo-parieto-hippocampal atrophy, progressive semantic and episodic memory deficits, and executive function declines [3]. Conversely, bvFTD involves fronto-insulo-temporal atrophy and social behavior changes such as disinhibition, apathy, compulsion, and impaired moral judgment [4]. Yet, their clinical differentiation in early stages is challenging due to several cognitive and behavioral overlaps [3,5]. From a linguistic perspective, these include difficulties with understanding and recounting daily situations, which patients from both populations narrate in a disorganized [6] and uninformative [7,8] fashion. Here, we investigate whether clinically motivated natural language processing (NLP) features can capture differential markers of each disorder.

Previous works have revealed connected speech alterations in both syndromes. People with AD have difficulties maintaining referential [9,10] and temporal [6] cohesion, which can affect global coherence. Also, they show reduced idea density and lexical diversity [11,12], leading to uninformative speech [13]. Early impairment in lexico-semantic abilities can also lead to word-finding delay [14,15], semantic paraphasias [14,16], and naming difficulties [17,18]. For their part, people with bvFTD exhibit reduced propositional content [7], poor idea organization [19], and increased superfluous content [20,21], alongside possible morphosyntactic deficits manifested as challenges in complex sentence comprehension and completion [22] and difficulty sequencing events [23]. Yet, linguistic markers have rarely been jointly examined in both populations, let alone by testing clinically grounded hypotheses in naturalistic routine descriptions. Such is the focus of this paper.

Daily situations are construed by identifying people, objects or other entities (expressed through nouns) engaged in actions or inner experiences (expressed through verbs) from an egocentric or exocentric perspective (via first or third person references) [24]. Persons with AD have been shown to produce fewer nouns (but not fewer verbs) than HCs during interviews, including questions about their experiences [25–27]. Suggestively, the same occurs in semantic dementia (another syndrome with primary semantic memory deficits) [28], but not in bvFTD [29]. This pattern aligns with models that propose that nouns are differentially subserved by temporal and temporo-parietal circuits [30,31] which are differentially affected in AD [32–34], while verbs would be critically underpinned by frontal/motor areas [30,35,36]. Conversely, bvFTD may involve abnormal perspective taking in social scenarios [37]. Patients are typified by inaccurate self-awareness and self-monitoring [38], and they favor a third-person perspective for self-representation [39]. Indeed, self-referential processing recruits prefrontal regions distinctly compromised in bvFTD [40]. These patterns, we surmise, may be reflected in a preference for third- over first-person pronouns in connected speech. In addition, evidence from more controlled tasks, such as verbal fluency, suggests that these syndromes may differ in their vocabulary navigation patterns, with AD (but not bvFTD) patients differing from HCs in using more frequent and otherwise more accessible words [41]. In sum, AD and bvFTD might be typified by specific anomalies in their linguistic expression of daily events.

Simple NLP tools are well-suited to test this conjecture. This approach can improve the characterization, diagnosis, and phenotyping of different neurodegenerative diseases [13], while having the potential to foster global equity in the fight against dementia [42]. More particularly, they can yield different insights into neurodegenerative disorders depending on the type of linguistic task analyzed. For instance, semi-spontaneous tasks, such as picture descriptions, seem well suited to evaluate semantic memory [13,43]. For their part, spontaneous tasks, such as unstructured interviews, better capture natural discourse profiles in terms of syntactic structure, coherence, and cohesion [9]. Among the latter, routine description tasks are useful to target the present study’s features, given their focus on objects and actions (typically described through nouns and verbs) that can be described from ego-centric or exo-centric perspectives.

Part-of-speech tagging tools can automatically identify nouns and distinguish them from other categories (e.g., verbs) [44]. Likewise, morphological tagging tools can discriminate between those coding egocentric (i.e., first-person markers, such as *I* and *my*) and exocentric reference (e.g., third-person markers, such as *she* and *her*) [44]. Also, word properties can be derived through fully automated algorithms [41]. Promisingly, given their simplicity, automaticity, and affordability, these tools could be leveraged in diverse clinical settings. However, despite the growth of NLP in dementia research [13,29,45], no study has tested this potential double dissociation, let alone combining inferential statistics and machine learning tools for group- and subject-level discrimination, respectively.

**Fig 1:**
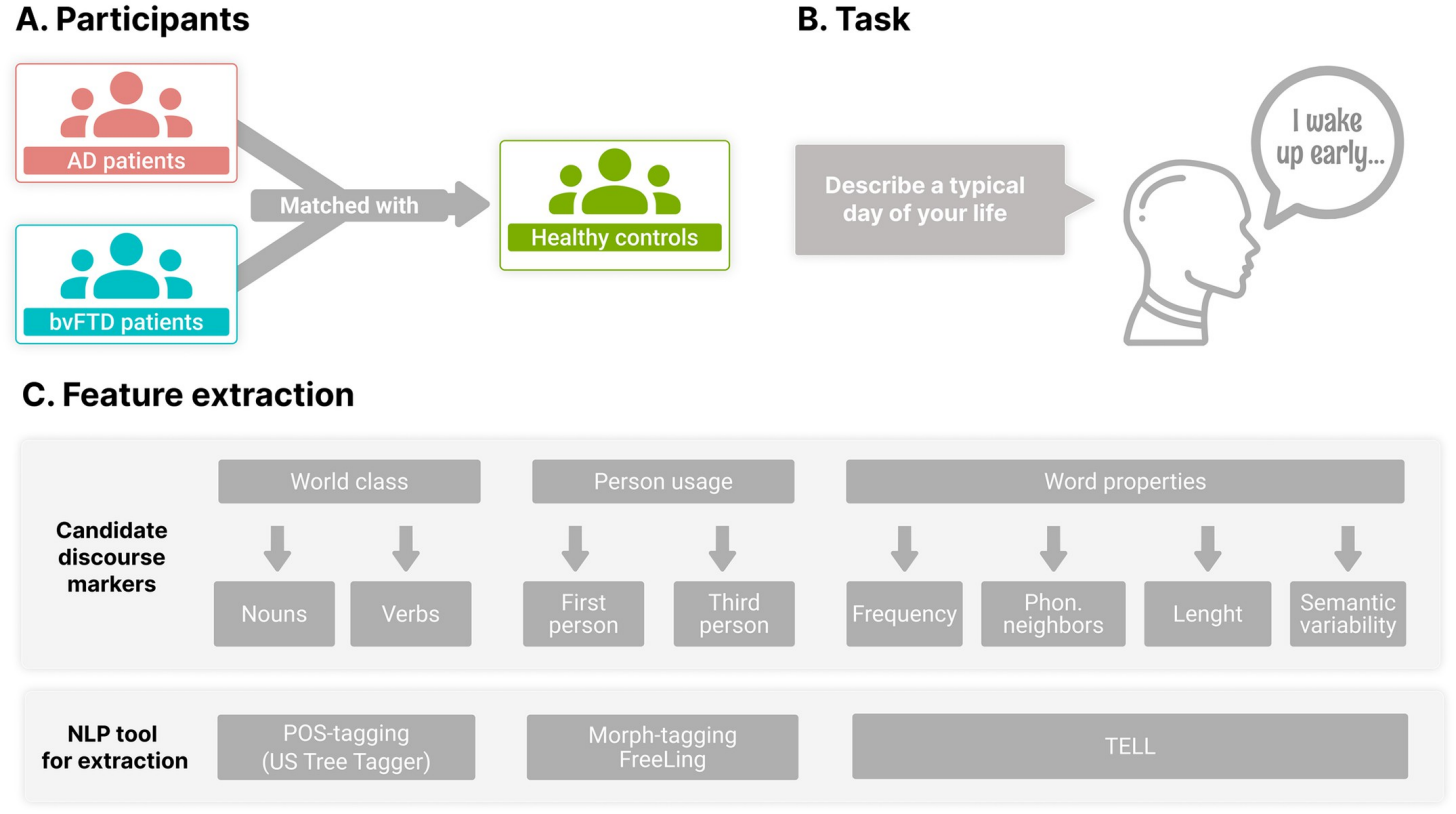
Study design. **(A)** Ninety-six subjects (32 with AD, 32 with bvFTD, and 32 healthy controls) performed a **(B)** routine description task. **(C)** Transcripts from their recordings were analyzed to examine predicted differences in their linguistic expression of situations, with a focus on (i) noun and verb ratios, derived through part-of-speech tagging; (ii) first- and third-person ratios, obtained via morphological tagging; and (iii) word properties (frequency, phonological neighborhood, length, semantic variability), captured through the TELL app. AD: Alzheimer’s disease; bvFTD: Behavioral variant frontotemporal dementia; NLP: Natural language processing; POS: Part of speech.

Here we examined whether persons with AD and bvFTD differ from HCs in their linguistic expression of daily events. We recorded participants as they described a day in their lives, transcribed their speech, and automatically calculated the proportion of nouns, verbs, first-person markers, and third-person markers (Fig 1). We performed a main study on a subset of participants, and reserved part of our sample for a generalizability test. First, we predicted that persons with AD, unlike those with bvFTD, would be selectively impaired in noun (but not verb) retrieval. Second, we hypothesized that persons with bvFTD, but not AD, would rely less on first-person reference and more on third-person reference. Third, we hypothesized that AD patients, unlike bvFTD patients, would employ more frequent words. Fourth, we anticipated that these features would offer good subject-level classification and robust generalizability to unseen samples in machine learning analyses. Finally, we explored whether these features were associated with overall cognitive impairment in each group. Briefly, with this approach, we aim to reveal novel automated markers of the two most prevalent forms of dementia.

## Materials and methods

### Participants

The study involved 96 native Spanish speakers, recruited in two centers from the ReDLat consortium. Our main analyses comprised 21 persons with AD, 21 persons with bvFTD, and 21 HCs (Fig 1A), reaching a power of 0.81 (S1 File). The remaining participants (11 with AD, 11 with bvFTD, 11 HCs) were used as a hold-out sample to test for generalizability in our machine learning analyses (S2 File). Patients were diagnosed by expert neurologists following NINCDS-ADRDA criteria for AD [46] and current clinical criteria for probable bvFTD [4]. Diagnoses were supported by an extensive neurological, neuropsychiatric, and neuropsychological examination [47] following unified procedures [48]. Persons with AD presented memory impairment and individuals with bvFTD exhibited socio-behavioral impairments verified by caregivers. Both groups showed general cognitive deficits, based on the Montreal Cognitive Assessment (MoCA) [49]; and executive dysfunction, as established through the INECO Frontal Screening (IFS) battery [50]. No patient reported a history of other neurological disorders, and none had primary linguistic deficits (as established through a neuropsychological interview, caregiver reports, and qualitative evaluation of conversational speech). HCs were cognitively preserved and functionally autonomous. None reported a background of neuropsychiatric disease or substance abuse. All participants had a normal or corrected-to-normal hearing, determined through a formal functionality survey. Each patient group was matched with HCs in terms of sex, age, and education. Demographic and neuropsychological details are shown in Table 1.

**Table 1:** Participant’s demographic and cognitive profiles.

	AD patients (*n* = 21)	bvFTD patients (*n* = 21)	Healthy controls (*n* = 21)	AD patients vs. healthy controls		bvFTD patients vs. healthy controls	
				Statistic	*p*-value	Statistic	*p*-value
**Sex (F:M)**	10:11	16:5	13:8	*χ*^*2*^ = 0.384	0.54	*χ*^*2*^ = 0.864	0.35^a^
**Age**	74.29 (6.54)	66.14 (6.92)	70.52 (10.26)	*F* = 1.52	0.23	*F* = 1.76	0.15^b^
**Years of Education**	12.71 (3.98)	14 (4.02)	12.76 (4.44)	*F* = 0.12	0.99	*F* = 0.96	0.53^b^
**MoCA**	14.53 (5.46)	17.67 (9.55)	24.91 (4.11)	*F* = 3.67	< 0.001	*F* = 2.63	< 0.05^b^
**IFS battery**	12.62 (6.29)	13.14 (6.33)	20.1 (4.37)	*F* = 3.98	< 0.001	*F* = 3.76	< 0.001^b^

Data is shown as mean (*SD*), except for sex. MoCA values were missing from 10 HCs, 6 AD patients, and 3 bvFTD patients; IFS values were missing for 1 HC, 6 AD patients, and 3 bvFTD patients. This was due to omissions during data tabulation or because the participants opted not to complete the tasks.

^(a)^ *p*-values were calculated via chi-squared test.

^(b)^ *p*-values were calculated via Dunnett’s test.

AD: Alzheimer’s disease; bvFTD: Behavioral variant frontotemporal dementia; MoCA: Montreal Cognitive Assessment; IFS: INECO Frontal Screening.

Recruitment for this study took place between May 11, 2021, and June 4, 2022. All participants provided written informed consent, documented by a researcher. The study was performed according to the Declaration of Helsinki and approved by the Ethics’ Committee of Universidad de Chile.

### Speech elicitation and transcription

Speech was elicited at the clinicians’ offices, after the neuropsychological evaluation. Participants were invited to describe a typical day in their lives (Fig 1B), since they woke up until they went to bed, with the following instruction: “Now you will describe your typical day in your life. Please describe everything you do since you wake up until you go to bed at night. Use as much detail as possible. For example, instead of saying ‘I make breakfast,’ tell me everything you do to make breakfast. Are you ready? Please speak at your usual speed, pitch, and volume.” Examiners were instructed to elicit between 1 and 2 minutes of speech from each participant. If a participant stopped talking before the 1-minute mark, examiners prompted them to continue speaking by saying “Tell me more.” Narrations were audio-recorded with high-end cell phones (sampling rate = 44.1 Hz, resolution = 16 bits), and saved as.wav files.

Audio-recordings were transcribed verbatim with Google speech-to-text software. Transcriptions were revised manually by Spanish-speaking neuropsychologists who were blind to group- and protocol-specific information. They were all specialized in language testing and followed reported procedures [51] including use of standard punctuation norms from Royal Spanish Academy (http://www.rae.es/) and allocation of full stops based on grammatical criteria. The very few instances of unintelligible words were discarded from the transcripts and analyses. Prior to feature extraction, all transcripts were tokenized (i.e., divided into smaller units apt for NLP and machine learning analysis) using Freeling 4.2 [44], without removing stop words (textual elements other than content words, including dates, punctuation markers, determiners, adpositions, conjunctions, interjections, numbers). Texts were not lemmatized (i.e., converted to their base form upon removing inflectional morphemes), as this would impede the identification of first- and third-person markers. Filled pauses, hesitations, and false starts were transcribed fully, even though they were excluded from analyses as our hypotheses focused on specific word categories. Importantly, transcribed strings that did not represent full words were omitted from analysis even if they belonged to our categories of interest—e.g., an interrupted noun, such as ‘*hospi*’ in *After lunch*, *I go to the hospi… rather*, *to the clinic*.

### Feature extraction

#### Automated estimation of word class usage

Nouns and verbs (Fig 1C) were automatically identified via FreeLing’s POS-tagger [44]. Specifically, based on a standard trigram hidden Markov model, it replaces each token by its lexical category (namely, nouns, verbs, adjectives, adverbs, determiners, pronouns, conjunctions and adpositions)–a context-sensitive task that FreeLing achieves with an accuracy of 95% [44]. The ratios of nouns and verbs per participant were calculated by reference to the total number of words, including stop words.

#### Automated estimation of person usage

First- and third-person usage (Fig 1C) was coded via FreeLing’s morphological tagging module [51]. This function assigns POS-specific morphological attributes to each token based on grammatical and/or semantic attributes of neighboring words, reaching an accuracy of 95% [44]. In particular, verbs and pronouns mark for the attribute ‘person’, which can take only one of three possible values: first, second, or third person (e.g., *camino*, *caminas*, *camina* [*I walk*, *you walk*, *s/he walks*, respectively]). For example, situations may be expressed through first- or third-person references, respectively signaled in bold and in underlined bold case in the next examples from our corpus.

*La señora se levant****a*** *primero*, *después* ***voy yo***.
[*The lady gets up first*, *and then I do so*]
***Mi*** *hijo lleg****a*** *y entonces* ***me*** *dej****a*** *las llaves y dic****e*** *que si quier****o me*** *dej****a*** *las puertas abiertas*.
[*My son arrives and then he gives me the keys and tells me that*, *if I want*, *he will open the doors for me*]

Of note, Spanish morphology and syntax offer explicit markers of these properties, as grammatical persons are unambiguously conveyed by the endings of inflected verbs in most tenses (e.g., first person is exclusively conveyed through the ending *-o* in present (e.g., *camino*), *-é* in past simple (e.g., *caminé*), and *-aré* in future simple (e.g., *caminaré*). Moreover, cases of morphological ambiguity (e.g., the imperfect past ending *-aba*, used for both first and third person) are resolved by intra-sentential person-verb agreement or inter-sentential reference (e.g., *Yo siempre caminaba*, where *yo* disambiguates *-aba* as a first-person marker).

Given the nature of the task and the focus of our hypothesis, we discarded all words with a second-person tag (these amounted to only 1.1% of all person-marked words in our corpus). The ratios of first- and third-person morphemes per participant were calculated by reference to the total number of person occurrences. Pronoun and verb morphemes related to the same event were considered separately (e.g., in the clause *Yo salgo*, the two first-person markers are counted individually towards our overall first-person marker ratio)–further considerations on this point are offered in the “Discussion” section.

#### Automated calculation of word properties

We used a novel automated pipeline [41,52], implemented in the TELL app [53], to capture objective lexico-semantic features across all content words (nouns, verbs, adjectives, adverbs) from each participant. We extracted four features from each content word (Fig 1C). Three of them were obtained through the EsPal database [54], namely: word *frequency* (logarithmic frequency per million), *phonological neighborhood* (number of words obtained by substituting, adding, or omitting a phoneme), and *length* (number of phonemes). The fourth was an NLP feature called semantic variability [41,52,55]. Based on a FastText model pre-trained with language-specific corpora, each text is mapped as a series of vectors, keeping the words’ sequence and omitting repetitions. Distances between adjacent vectors are stored into a time series. Semantic variability is computed as the variance of the text’s joint time series. When semantic distance across adjacent words is inconsistent, the text has high semantic variability.

#### Quality check

To ensure that the labels underlying all calculations were adequate, we asked a trained Spanish-speaking psychologist, specialized in language research, to perform a manual revision of all tags from 25% of the transcriptions in each group within our main study. The process showed that automated tags had an accuracy of 90.1%.

### Statistical analysis

#### Group-level comparisons via inferential statistics

Statistical comparisons were performed between subjects with AD and HCs, and between subjects with bvFTD and HCs, for two dependent variables: word class usage (noun ratio, verb ratio) and person usage (first-person ratio, third-person ratio). In each case, we performed 2x2 mixed ANOVAs, with group as a between-subjects factors (patients, HCs) and tag ratio as a within-subject factor (nouns and verbs, in the case of word class; first and third person, in the case of person usage). Post hoc comparisons were made through Tukey’s HSD tests. Moreover, each word property (frequency, phonological neighborhood, length, semantic variability) was compared between groups via a separate one-tailed independent samples *t*-tests. Alpha levels were set at *p* < 0.05. No participant was detected as an outlier in any dataset (at a threshold of 3 *SD*s from the sample’s mean). All results were corrected for multiple comparisons via the false discovery rate (FDR) metric. Analyses were run on Python 3 (via Pandas 1.3.2 and Pingouin 0.5.1 packages).

#### Subject-level classification via machine learning analysis

We utilized a support vector machine (SVM) classifier with a linear kernel to discriminate between patients in each group from HCs. This method models probabilities based on a decision boundary that maximizes the margin between the classes [56], yielding robust results in language and neuropsychological research on dementia [57]. Each classifier was trained using all linguistic features. The data were randomly split into five folds for stratified cross-validation, preserving the proportion of labels per group [58], where four folds were used for training and one fold was used for testing. The values of each feature were normalized using the min-max method [59]. AUC, accuracy, precision, recall, F1, and UAR scores were reported as mean and *SD* upon 1000 iterations with different random data partitions. Also, we calculated the contribution of each feature to overall classification, considering the absolute values of each feature’s coefficient in a feature importance analysis. All analyses were performed using Python 3.9 and the Scikit-learn (https://scikit-learn.org/) package. ROC curve plots and feature importance graphs were created using Seaborn Python’s library [60] and Ggplot R’s library [61].

#### Generalizability tests

To test the generalizability of the machine learning models, we replicated our approach by (i) training each binary classifier (AD patients vs. HCs; bvFTD patients vs. HCs) with all participants from our main study and then (ii) testing them on a hold-out set composed of different participants (11 AD patients, 11 bvFTD patients, 11 HCs). These new groups were socio-demographically matched with each other and relative to the participants of the main study (S2 File). This analysis employed the same pipeline of the machine learning analysis in our main study.

#### Correlations between connected speech features and cognitive status

We examined whether our target features were associated with patients’ cognitive status, as captured by the MoCA and the IFS battery. Correlations were based on Pearson’s coefficients, corrected for multiple comparisons via FDR, and performed on GraphPad Prism^®^ (v 6.01).

## Results

### Speech elicitation

The mean number of words produced by HCs (304.14) did not differ significantly from that of individuals with AD (238.57; *t*_(40)_ = 1.28, *p* = 0.21, *d* = 0.40) or bvFTD (253.95; *t*_(40)_ = 1.03, *p* = 0.31, *d* = 0.32). Instances of unintelligible words, due to recording issues and/or speech errors, were discarded from the transcripts and analyses. These represented fewer than 0.01% of words in each group, and they did not differ significantly between AD patients and HCs (*t* = 0.8297, *p* = 0.41) or between bvFTD patients and HCs (*t* = 0.4515, *p* = 0.65).

### Word class usage

**Fig 2:**
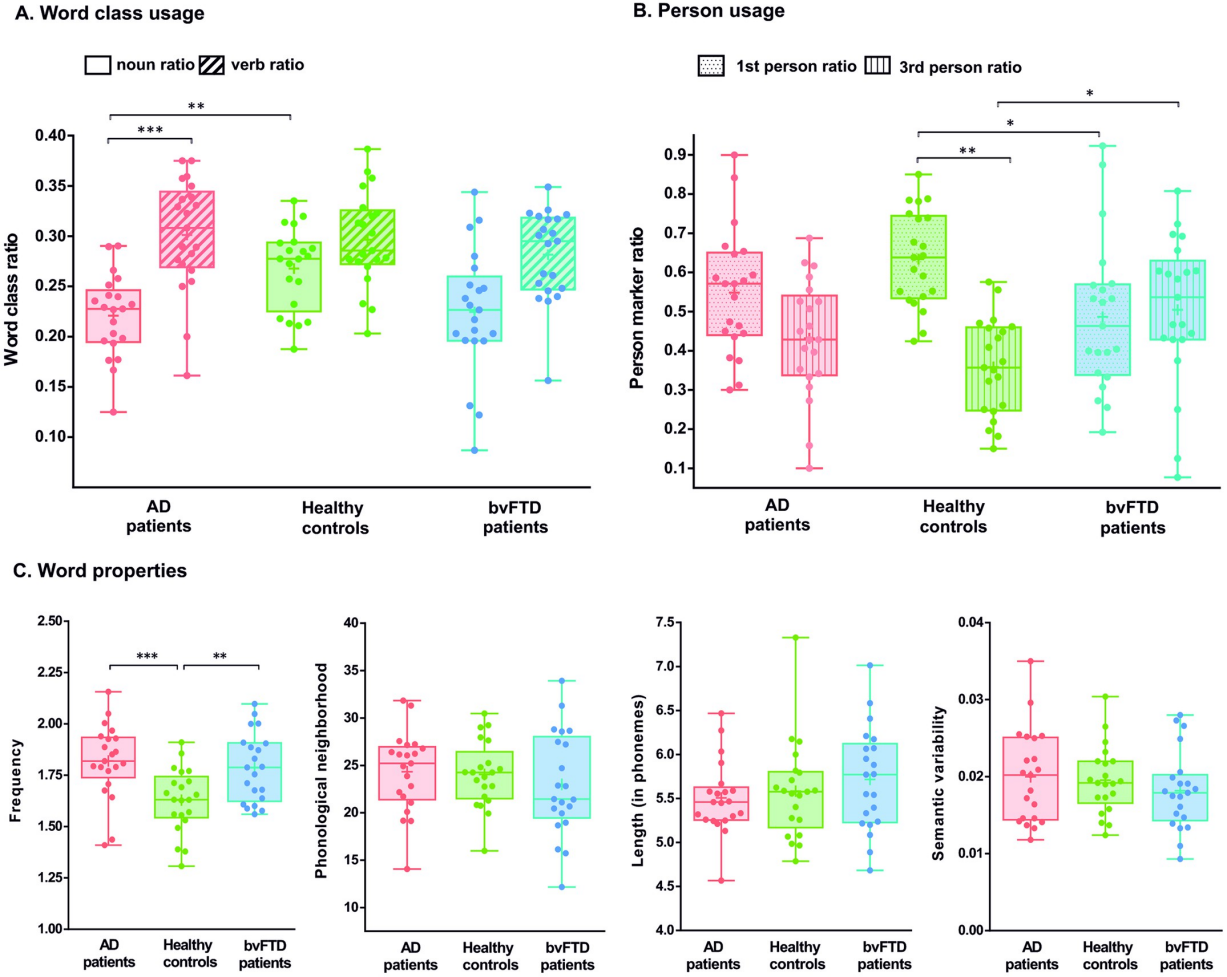
Between-group comparisons of connected speech features. **(A)** Noun ratio was lower for AD (but not bvFTD) patients compared with HCs. No between-group differences emerged for verb ratio. **(B)** BvFTD (but not AD) patients produced significantly fewer first-person and more third-person markers than HCs. **(C)** Both patient groups produced more frequent content words than did HCs, there being no between-group differences in other word properties (phonological neighborhood, length, semantic variability). In all cases the mean is indicated by the cross. For ease of visualization, brackets show only significant pairwise comparisons from significant interaction effects, performed via Tukey’s HSD test. The number of asterisks denote the alpha threshold of the effect (* = *p* < 0.05, ** = *p* < 0.01, *** = *p* < 0.001). AD: Alzheimer’s disease; bvFTD: Behavioral variant frontotemporal dementia.

The comparison between subjects with AD and HCs (Fig 2A) revealed a non-significant effect of group [*F*(1,40) = 3.99, *p*FDR = 0.125, np^2^ = 0.09] and a significant effect of word class [*F*(1,40) = 30.89, *p*FDR = 0.001, np^2^ = 0.43]. Crucially, the interaction between both factors was significant [*F*(1,40) = 6.97, *p*FDR = 0.024; np^2^ = 0.15]. Post hoc analysis, via Tukey’s HSD test (*df* = 40, *MSE* = 0.014), showed that, relative to HCs, persons with AD produced fewer nouns (pFDR = 0.024, *d* = 1.145) and a similar proportion of verbs (*p*FDR = 0.990, *d* = -0.95). Also, while the proportion of both word classes was similar in HCs (*p*FDR = 0.240, *d* = 0.65), persons with AD produced fewer nouns than verbs (*p*FDR = 0.008, *d* = 1.64).

As regards the comparison between subjects with bvFTD and HCs (Fig 2A), we found a significant group effect [*F*(1,40) = 8.98, *p*FDR = 0.020, np^2^ = 0.18], indicating fewer items (regardless of word class) in the patients. We also found a significant word class effect [*F*(1,40) = 12.98, *p*FDR = 0.001, np^2^ = 0.25], revealing a lower ratio of nouns than verbs across groups. The interaction between both factors was not significant [*F*(1,40) = 1.42, *p*FDR = 0.240, np^2^ = 0.03]. For full details, see S3 File.

### Person usage

The comparison between subjects with AD and HCs (Fig 2B) yielded a significant effect of person [*F*(1,40) = 20.79, *p*FDR = 0.001, np^2^ = 0.34], with a lower proportion of third-person than first-person items across groups. Neither the main effect of group [*F*(1,40) = 2.82, *p*FDR = 0.202, np^2^ = 0.07] nor the interaction between group and person [*F*(1,40) = 3.43, *p*FDR = 0.098, np^2^ = 0.08] were significant.

Conversely, comparisons between subjects with bvFTD and HCs (Fig 2B) revealed a non-significant effect of group [*F*(1,40) = 0.06, *p*FDR = 0.815, np^2^ = 0.002] and a significant effect of person [*F*(1,40) = 6.78, *p*FDR = 0.013, np^2^ = 0.14]. Crucially, a significant interaction emerged between both factors [*F*(1,40) = 8.77, *p*FDR = 0.020, np^2^ = 0.18]. Post hoc analysis, via Tukey’s HSD test (*df* = 40, *MSE* = 0.451), revealed that, relative to HCs, persons with bvFTD produced a significantly lower proportion of first-person items (*p*FDR = 0.042, *d* = -0.91) and a significantly greater proportion of third-person items (*p*FDR = 0.042, *d* = 0.915). Also, HCs relied significantly more on first- than third-person items (*p*FDR = 0.008, *d* = -2.239), but no such difference was observed in persons with bvFTD (*p*FDR = 0.999, *d* = 0.093). For full details, see S3 File.

### Word properties

Word property analyses revealed that both patient groups produced more frequent content words than HCs [AD: *t*(40) = 3.682, *p*FDR = 0.001, *d* = 1.13; bvFTD: *t*(40) = -3.177, *p*FDR = 0.003, *d* = -0.98]. No further word property yielded significant differences in either patient group (all *p*FDR values > 0.36). For full details, see S3 File.

### Subject-level classification

**Fig 3:**
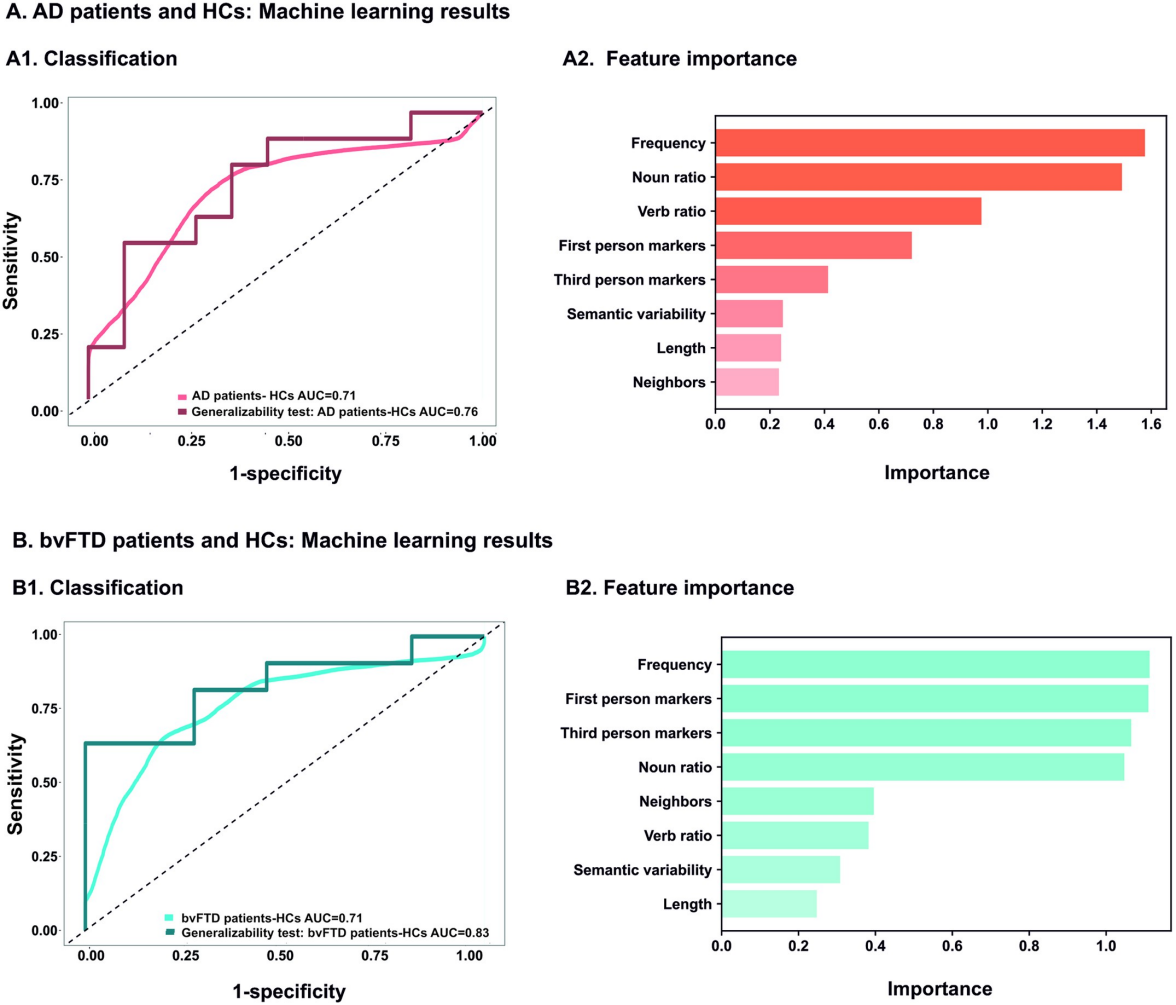
ROC curves with AUC scores. Support vector machines were used to classify between each patient group and HCs over 1000 iterations, using all linguistic features together. **(A)** Classification between AD patients and HCs reached AUCs of 0.71 and 0.76 in the main study and the generalizability test, respectively. These outcomes were driven by word frequency and noun ratio. **(B)** Classification between bvFTD patients and HCs reached AUCs of 0.71 and 0.83 in the main study and the generalizability test, respectively. These outcomes were driven by word frequency, first-person markers, third-person markers, and noun ratio. AD: Alzheimer’s dementia; AUC: Area under the receiver operating characteristic curve; bvFTD: Behavioral variant frontotemporal dementia; HCs: Healthy controls; SVM: Support vector machines.

Joint analysis of all features yielded good patient identification in both cases (persons with AD vs. HCs: AUC = 0.71 ± 0.14; persons with bvFTD vs. HCs: AUC = 0.71 ± 0.14). Classification between persons with AD and HCs was mainly driven by word frequency and noun ratio, surpassing the weight of the other features by at least 50%. Conversely, classification between persons with bvFTD and HCs was similarly driven by word frequency, first-person markers, third-person markers, and noun ratio, surpassing every other feature by over 100%. ROC curves with AUC scores and feature importance rankings are shown in Fig 3. For full details, see S4 File.

### Generalizability tests

Generalizability tests (Fig 3) revealed that results remained robust, and actually improved, when training classifiers with our main study’s participants and testing them on entirely separate samples (persons with AD vs. HCs: AUC = 0.76; persons with bvFTD vs. HCs: AUC = 0.83). For full details, see S4 File.

### Correlations with overall cognitive status

Correlations between connected speech features and indices of cognitive status (MoCA scores) and executive functions (IFS scores) were not significant in either patient group. See Table 2.

**Table 2:** Correlations between connected speech features and cognitive indices.

	Groups	Cognitive test	*p*-value	*R*
**Noun ratio**	AD patients vs. HCs	MoCA	0.06	0.54
		IFS	0.17	0.39
	bvFTD patients vs. HCs	MoCA	0.06	0.52
		IFS	0.48	0.25
**Verb ratio**	AD patients vs. HCs	MoCA	0.96	0.01
		IFS	0.46	-0.29
	bvFTD patients vs. HCs	MoCA	0.62	0.20
		IFS	0.84	0.08
**First person markers**	AD patients vs. HCs	MoCA	0.91	0.04
		IFS	0.84	0.08
	bvFTD patients vs. HCs	MoCA	0.84	0.14
		IFS	0.84	0.07
**Third person markers**	AD patients vs. HCs	MoCA	0.84	-0.07
		IFS	0.84	-0.08
	bvFTD patients vs. HCs	MoCA	0.84	-0.12
		IFS	0.87	-0.05
**Frequency**	AD patients vs. HCs	MoCA	0.28	-0.40
		IFS	0.42	-0.31
	bvFTD patients vs. HCs	MoCA	0.59	-0.22
		IFS	0.51	-0.23
**Phonological neighborhood**	AD patients vs. HCs	MoCA	0.51	-0.27
		IFS	0.48	-0.25
	bvFTD patients vs. HCs	MoCA	0.54	-0.23
		IFS	0.84	0.06
**Length**	AD patients vs. HCs	MoCA	0.84	0.08
		IFS	0.48	0.26
	bvFTD patients vs. HCs	MoCA	0.84	0.12
		IFS	0.96	0.02
**Semantic variability**	AD patients vs. HCs	MoCA	0.84	-0.14
		IFS	0.51	-0.22
	bvFTD patients vs. HCs	MoCA	0.64	0.19
		IFS	0.84	0.08

All analyses were performed via Spearman’s correlations, corrected by the false discovery rate metric. AD: Alzheimer’s disease; bvFTD: Behavioral variant frontotemporal dementia; HCs: Healthy controls; IFS: INECO Frontal Screening battery; MoCA: Montreal Cognitive Assessment.

## Discussion

We used NLP tools to examine differential markers of AD and bvFTD in patients’ expression of daily events. Relative to HCs, only persons with AD showed a reduced proportion of nouns (but not verbs), pointing to a category-specific anomaly. Conversely, only persons with bvFTD used fewer first person and more third person references than HCs, indicating more exocentric discourse. These features offered good subject-level classification in both groups, and they did not correlate with patients’ cognitive status. We elaborate on these findings below.

Word class analyses revealed differential anomalies in each patient group. Compared with HCs, persons with AD produced significantly fewer nouns and a similar proportion of verbs. The same pattern was observed in younger AD cohorts when asked open-ended self-referential questions—using both manual and automated word-class coding [25,26]. Thus, while AD patients may also exhibit distinct difficulties with verbs in structured tasks [62], noun retrieval may be specifically compromised in their connected speech. Importantly, this selective pattern was not observed in bvFTD patients, who, relative to HCs, produced lower proportions of *both* nouns *and* verbs. These results contrast with previous bvFTD studies reporting non-significant differences in either category relative to HCs [29] and greater difficulties with verbs than nouns compared with AD patients [63]. Yet, they all converge in the absence of differential deficits for nouns, as observed here for AD. Thus, selective reductions in noun production during spontaneous speech might afford differential markers of this dementia type.

In line with previous works [30,64–66], this selective pattern might reflect the distinct reliance of noun information on declarative memory circuits compromised by AD. Indeed, neuroimaging, neurostimulation, and lesion studies show that object naming and knowledge hinge on temporal regions [30] typically compromised in AD, such as the middle and inferior temporal lobe [32]. These regions, indeed, have been proposed to integrate information from different sensory streams, as required to adequately construe noun referents in semantic memory [31]. Although noun processing may also involve a wider distributed network spanning other regions [30], these alterations would lead AD patients to single out fewer nouns in their depiction of daily events.

Conversely, person usage results revealed a different pattern. While HCs and AD patients relied significantly more on first-person markers, bvFTD patients exhibited the opposite picture. Previous works with bvFTD patients have reported reduced insight into their own behavioral changes [67] as well as impairments in recent and remote self-related recollections, including specific and contextually rich autobiographical memories [37]. Moreover, persons with bvFTD have been shown to prefer an observer perspective when retrieving personal events, together with decreased capacity to relive sensory and/or affective details [39]. Of note, the absence of this distinction in AD aligns with previous works showing same proportions of both first- and third-person pronouns relative to HCs [68] and preserved semantic knowledge of patients’ personal history and sense of self [37]. In sum, bvFTD might be differentiated from AD by their tendency to depersonalize self-related narratives.

These results align with situated views of language disruptions, which posit that specific cognitive and socio-affective deficits are reflected in germane linguistic categories [69,70]. Specifically, disruption of neurocognitive systems mediating self-awareness and perspective taking would lead to reduced self-reference, leading to exocentric (third person) linguistic references to construe daily events. Indeed, self-attention [71] as well as perspective taking skills [72] have been linked to fronto-temporo-parietal hubs that are abnormally connected in bvFTD [73]. Reduced reliance on first-person markers in bvFTD, then, might be a recapitulation of more basic self-processing deficits in non-verbal domains.

Note that, in our analyses, person markers duplicated in pronoun-verb tandems were counted twice. This aimed to capture person usage in its full scope given the morpho-syntactic properties of Spanish. As a pro-drop language, Spanish allows for pronoun dropping without compromising a clause’s grammatical integrity, given that key information to establish a verb’s referent (person and number) is coded in its desinence. Yet, pronouns may well be (and are often) inserted even if the verb provides such anchorage, be it because of rhetorical (e.g., emphatic), cohesive (e.g., referential) or subjective (e.g., idiolectal) reasons. Therefore, separate counting of person markers in pronoun-verb dyads may capture relevant subject-level information, and, in any case, this premise operated equally on both first- and third-person markers, further reducing the possibility of bias in our results. More generally, this issue illustrates the importance of tackling language-specific phenomena when pursuing NLP markers of dementia, as noted in recent calls [42].

Interestingly, word property analyses did not yield syndromic differentiations. Both AD and bvFTD patients used significantly more frequent words than HCs. This differs from a previous application of the same automated pipeline, which revealed a preference for higher frequency words only in AD patients [41]. Such discrepancy might be partly explained by task demands, as Ferrante et al.’s study was based on highly controlled verbal fluency tasks. Indeed, such tasks focally target semantic memory mechanisms (which are differentially impaired in AD [46]), whereas routine description, being a spontaneous speech task [13], requires an integration of multiple context-sensitive processes that might reduce the cognitive resources available for vocabulary navigation in both syndromes—indeed, some of the non-significant features, such as semantic variability, seem useful to capture deficits in both patient groups via controlled word-level tasks [41,52,55]. Further research involving different tasks would be needed to directly test this possibility.

Additional insights come from machine learning results. Joint analysis of all features yielded good classification of both AD and bvFTD patients relative to HCs, with generalizability tests reaching AUCs of 0.76 and 0.83, respectively. Similar classification outcomes were obtained in previous machine learning studies, although these varied in the clinical grounding of their target features [8,52,74]. Of note, generalizability tests results surpassed those of our main study. This is probably because the training set of the generalizability analyses employed the entirety of the main study participants, increasing training information by 20%. This finding invites replications on even larger samples, and, more generally, reinforces the role of fine-grained, hypothesis-driven NLP metrics as markers that generalize across individual patients [52]. Indeed, linguistic features were not significantly correlated with MoCA scores in either group, suggesting that they were not particularly affected in patients with higher cognitive severity. Interestingly, too, while word frequency emerged as a top discriminatory feature for both groups, this was closely followed by noun ratio in AD and by first- and third-person markers (alongside noun ratio) in bvFTD, surpassing every other feature. This reinforces the differential importance of noun processing and perspective-taking markers for each syndrome, while underscoring the value of multivariate linguistic analyses for capturing sensitive signatures of dementia [29,52].

While other NLP works have targeted broad collections of linguistic features in a data-driven fashion [8,45], our study underscores the utility of hypothesis-led assessments. The analysis of features related to each disorder’s distinct neuropsychological profile can increase interpretability and specificity, maximizing clinical utility [52,75]. Even with only a few variables, our study captures distinct alterations in each dementia type. In addition, our approach rests exclusively on automated tools. As such, it involves low costs and does not require highly specialized clinical staff, often limited in vulnerable world regions [76]. Briefly, then, further applications of this framework could aid the quest for scalable and equitable markers of dementia [41,42,53,77].

## Limitations and avenues for further research

Our study is not without limitations. First, although our study was adequately powered and replicable NLP results have been obtained with similar sample sizes [52,78], it would be vital to test our approach with more participants. Second, our dataset lacked standard measures of person/object knowledge and perspective taking, precluding analyses of these variables relative to our target linguistic features. Future studies with such tasks would allow examining potential key drivers of the markers identified herein. Third, quantification of person markers requires specific methodological decisions guided by language-specific properties. Our earlier discussion of pronoun usage in Spanish underscores this issue and invites further questions on how such patterns might be affected by contextual factors (e.g., task demands), age (e.g., early and late-onset patients), and socioeconomic variables (e.g., education level). Future studies should expand our approach by interpolating these and other relevant variables. Fourth, while our study focused on a single task relevant to our target features (routine description), future studies should include others (e.g., story retellings, other spontaneous narratives) to establish how informative such features prove when connected speech is elicited under different processing conditions. For example, while routine description is an autobiographical memory task, story retelling may be based on non-self-referential material, and, when based on written auditory prompts, it can substantially increase memory and executive load. Future studies, then, could examine whether specific NLP markers are being optimally leveraged depending on task demands—for relevant insights, see Boschi et al. [13]. Fifth, new studies should include neuroimaging tools to reveal anatomo-functional signatures of each disorder’s linguistic alterations. Finally, cross-cultural replications would be important to ascertain whether the observed patterns generalize across different languages [42].

## Conclusions

This work suggests that AD and bvFTD exhibit distinct alterations in their expression of daily events. While AD patients might be typified by reduced reliance on nouns, persons with bvFTD would favor exocentric (third person) perspective on events. These patterns can be captured automatically with NLP tools, which are objective, inexpensive, and scalable. Future works should further test the clinical utility of digital language markers for dementia assessments.

## Supporting information

S1 File Power estimation. (DOCX)

S2 File Hold-out samples’s demographics. **S1 Table**. Sociodemographic profile of the hold-out samples used for generalizability tests. **S2 Table**. Sociodemographic profile of the entire samples, collapsing the main study and holdout samples.
(DOCX)

S3 File Full statistical results. **S3 Table**. Word class usage: Full statistical results. **S4 Table**. Person usage: Full statistical results. **S5 Table**. Word properties: Full statistical results.
(DOCX)

S4 File Full machine learning results. **S6 Table**. Main study: Additional performance metrics for the classification analyses. **S7 Table**. Generalizability tests: Additional performance metrics for the classification analyses.
(DOCX)
