# Supplementary material for: Automated free speech analysis reveals distinct markers of Alzheimer’s and frontotemporal dementia
Source: PLoS One. 2024 Jun 6;19(6):e0304272. doi: 10.1371/journal.pone.0304272 (PMC11156374; doi:10.1371/journal.pone.0304272)
Supplement: S2 File — S1 Table. Sociodemographic profile of the hold-out samples used for generalizability tests. S2 Table. Sociodemographic profile of the entire samples, collapsing the main study and holdout samples. (DOCX) [file pone.0304272.s002.docx]

***Supporting information 2. Hold-out samples’ demographics***

**Automated free speech analysis reveals distinct markers of Alzheimer’s and frontotemporal dementia**

Pamela Lopes da Cunha,^1,2,¶^ Fabián Ruiz,^1,¶^ Franco Ferrante,^1,2,3^

Lucas Federico Sterpin,^1^ Agustín Ibáñez,^1,4,5^ Andrea Slachevsky,^6,7,8,9^

Diana Matallana,^10,11,12^ Ángela Martínez-R,^13^ Eugenia Hesse,^1,14^ Adolfo M. García,^1,4,5,15,*^

^1^ Cognitive Neuroscience Center, Universidad de San Andrés, Victoria, Buenos Aires, Argentina

^2^ Consejo Nacional de Investigaciones Científicas y Técnicas (CONICET), Ciudad Autónoma de Buenos Aires, Argentina

^3^ Facultad de Ingeniería, Universidad de Buenos Aires (FIUBA), Ciudad Autónoma de Buenos Aires, Argentina

^4^ Latin American Brain Health (BrainLat) Institute, Universidad Adolfo Ibáñez, Santiago, Peñalolén, Región Metropolitana, Chile

^5^ Global Brain Health Institute, University of California San Francisco, San Francisco, California, United States; and Trinity College Dublin, Dublin, Ireland

^6^ Neuropsychology and Clinical Neuroscience Laboratory (LANNEC), Physiopathology Program – Institute of Biomedical Sciences (ICBM), Neuroscience and East Neuroscience Departments, Faculty of Medicine, University of Chile, Santiago, Chile.

^7^ Geroscience Center for Brain Health and Metabolism (GERO), Providencia, Santiago, Chile

^8^ Memory and Neuropsychiatric Center (CMYN), Neurology Department, Hospital del Salvador and Faculty of Medicine, University of Chile, Providencia, Santiago, Chile

^9^ Servicio de Neurología, Departamento de Medicina, Clínica Alemana-Universidad del Desarrollo, Las Condes, Región Metropolitana, Chile

^10^ Instituto de Envejecimiento, Departamento de Psiquiatría (Programa PhD Neurociencias), Facultad de Medicina, Pontificia Universidad Javeriana, Bogotá, Colombia

^11^ Centro de Memoria y Cognición, Intellectus, Hospital Universitario San Ignacio Bogotá, San Ignacio, Colombia

^12^ Departamento de Salud Mental, Hospital Universitario Santa Fe de Bogotá, Bogotá, Colombia

^13^ Escuela de Medicina y Ciencias de la Salud, Universidad del Rosario, Bogotá, Colombia

^14^ Departamento de Matemática, Universidad de San Andres, Victoria, Buenos Aires, Argentina

^15^ Departamento de Lingüística y Literatura, Facultad de Humanidades, Universidad de Santiago de Chile, Estación Central, Santiago, Chile

***Corresponding author:**

E-mail: adolfo.garcia@gbhi.org

**Table S1.** Sociodemographic profile of the hold-out samples used for generalizability tests.

|  | **AD**  **patients**  **(*n* = 11)** | **bvFTD**  **patients**  **(*n* = 11)** | **Healthy**  **controls**  **(*n* = 11)** | **AD patients**  **vs. healthy controls** | | **bvFTD patients**  **vs. healthy controls** | |
| --- | --- | --- | --- | --- | --- | --- | --- |
|  |  |  |  | **Statistic** | ***p*-value** | **Statistic** | ***p*-value** |
| **Sex (F:M)** | 8:3 | 6:5 | 8:3 | *χ²* = 0.00 | 1.00 | *χ²* = 0.79 | 0.38 |
| **Age** | 64.82  (6.43) | 73.18  (4.83) | 68.46  (8.40) | *t* = -1.27 | 0.35 | *t* = 1.66 | 0.19 |
| **Years of**  **Education** | 13.90  (2.77) | 13.70  (2.79) | 14.82  (2.68) | *t* = -0.77 | 0.67 | *t* = -0.93 | 0.56 |
| **MMSE** | 22.70  (3.83) | 23.00  (5.37) | 29.36  (1.03) | *t* = -3.96 | < 0.001 | *t* = -3.87 | < 0.01 |
| Data shown as mean (*SD*). Sex distributions were compared between groups via chi-squared tests. Continuous variables were compared between groups via unpaired *t*-tests. AD: Alzheimer’s disease; bvFTD: behavioral variant frontotemporal dementia; MMSE: Mini-Mental State Examination. | | | | | | | |

**Table S2.** Sociodemographic profile of the entire samples, collapsing the main study and holdout samples.

|  | **AD**  **patients**  **(*n* = 32)** | **bvFTD**  **patients**  **(*n* = 32)** | **Healthy**  **controls**  **(*n* = 132)** | **AD patients**  **vs. healthy controls** | | **bvFTD patients**  **vs. healthy controls** | |
| --- | --- | --- | --- | --- | --- | --- | --- |
|  |  |  |  | **Statistic** | ***p*-value** | **Statistic** | ***p*-value** |
| **Sex (F:M)** | 18:14 | 22:9 | 21:11 | *χ²* = 0.59 | 0.44 | *χ²* = 0.21 | 0.65 |
| **Age** | 71.03  (7.86) | 68.56  (7.07) | 69.81  (9.56) | *t* = 0.59 | 0.78 | *t* = -0.61 | 0.77 |
| **Years of**  **Education** | 13.10  (3.63) | 13.90  (3.63) | 13.47  (4.00) | *t* = -0.40 | 0.90 | *t* = 0.46 | 0.86 |
| Data shown as mean (*SD*). Sex distributions were compared between groups via chi-squared tests. Continuous variables were compared between groups via unpaired *t*-tests. AD: Alzheimer’s disease; bvFTD: behavioral variant frontotemporal dementia. | | | | | | | |
