# Supplementary material for: Automated free speech analysis reveals distinct markers of Alzheimer’s and frontotemporal dementia
Source: PLoS One. 2024 Jun 6;19(6):e0304272. doi: 10.1371/journal.pone.0304272 (PMC11156374; doi:10.1371/journal.pone.0304272)
Supplement: S3 File — S3 Table. Word class usage: Full statistical results. S4 Table. Person usage: Full statistical results. S5 Table. Word properties: Full statistical results. (DOCX) [file pone.0304272.s003.docx]

***Supporting information 3. Full statistical results***

**Automated free speech analysis reveals distinct markers of Alzheimer’s and frontotemporal dementia**

Pamela Lopes da Cunha,^1,2,¶^ Fabián Ruiz,^1,¶^ Franco Ferrante,^1,2,3^

Lucas Federico Sterpin,^1^ Agustín Ibáñez,^1,4,5^ Andrea Slachevsky,^6,7,8,9^

Diana Matallana,^10,11,12^ Ángela Martínez-R,^13^ Eugenia Hesse,^1,14^ Adolfo M. García,^1,4,5,15,*^

^1^ Cognitive Neuroscience Center, Universidad de San Andrés, Victoria, Buenos Aires, Argentina

^2^ Consejo Nacional de Investigaciones Científicas y Técnicas (CONICET), Ciudad Autónoma de Buenos Aires, Argentina

^3^ Facultad de Ingeniería, Universidad de Buenos Aires (FIUBA), Ciudad Autónoma de Buenos Aires, Argentina

^4^ Latin American Brain Health (BrainLat) Institute, Universidad Adolfo Ibáñez, Santiago, Peñalolén, Región Metropolitana, Chile

^5^ Global Brain Health Institute, University of California San Francisco, San Francisco, California, United States; and Trinity College Dublin, Dublin, Ireland

^6^ Neuropsychology and Clinical Neuroscience Laboratory (LANNEC), Physiopathology Program – Institute of Biomedical Sciences (ICBM), Neuroscience and East Neuroscience Departments, Faculty of Medicine, University of Chile, Santiago, Chile.

^7^ Geroscience Center for Brain Health and Metabolism (GERO), Providencia, Santiago, Chile

^8^ Memory and Neuropsychiatric Center (CMYN), Neurology Department, Hospital del Salvador and Faculty of Medicine, University of Chile, Providencia, Santiago, Chile

^9^ Servicio de Neurología, Departamento de Medicina, Clínica Alemana-Universidad del Desarrollo, Las Condes, Región Metropolitana, Chile

^10^ Instituto de Envejecimiento, Departamento de Psiquiatría (Programa PhD Neurociencias), Facultad de Medicina, Pontificia Universidad Javeriana, Bogotá, Colombia

^11^ Centro de Memoria y Cognición, Intellectus, Hospital Universitario San Ignacio Bogotá, San Ignacio, Colombia

^12^ Departamento de Salud Mental, Hospital Universitario Santa Fe de Bogotá, Bogotá, Colombia

^13^ Escuela de Medicina y Ciencias de la Salud, Universidad del Rosario, Bogotá, Colombia

^14^ Departamento de Matemática, Universidad de San Andres, Victoria, Buenos Aires, Argentina

^15^ Departamento de Lingüística y Literatura, Facultad de Humanidades, Universidad de Santiago de Chile, Estación Central, Santiago, Chile

***Corresponding author:**

E-mail: adolfo.garcia@gbhi.org

**Table S3.** Word class usage: Full statistical results.

|  | **Groups** | **Effect** | ***F*** | ***p*-value** | **ƞp^2^** | **Pairwise comparisons^#^** | | |
| --- | --- | --- | --- | --- | --- | --- | --- | --- |
|  |  |  |  |  |  | **Comparison** | ***p*-value** | **Cohen’s *d*** |
| **Word**  **class usage** | AD patients  vs. HCs | Group  Word class  Interaction | 3.99  30.89  6.97 | .156  .001  .024 | .091  .436  .148 | AD noun  vs. HC noun | 0.024 | 1.145 |
|  |  |  |  |  |  | AD verb  vs. AD noun | 0.008 | 1.64 |
|  |  |  |  |  |  | HC verb  vs. HC noun | 0.240 | 0.650 |
|  |  |  |  |  |  | AD verb  vs. HC verb | 0.990 | -0.95 |
|  | bvFTD  patients  vs. HCs | Group  Word class  Interaction | 8.98  12.98  1.42 | .020  .001  .240 | .183  .245  .034 | ------ | ------ | ------ |
| # Pairwise comparisons are shown when the interaction effect is significant. All *p*-values are FDR corrected. AD: Alzheimer’s disease; bvFTD: behavioral variant frontotemporal dementia; HCs: healthy controls. | | | | | | | | |

**Table S4.** Person usage: Full statistical results.

|  | **Groups** | **Effect** | ***F*** | ***p*-value** | **ƞp^2^** | **Pairwise comparisons^#^** | | |
| --- | --- | --- | --- | --- | --- | --- | --- | --- |
|  |  |  |  |  |  | **Comparison** | ***p*-value** | **Cohen’s *d*** |
| **Person usage** | AD patients  vs. HCs | Group  Person  Interaction | 2.83  20.79  3.43 | .242  .001  .098 | .066  .342  .079 | ------ | ------ | ------ |
|  | bvFTD patients vs. HCs | Group  Person  Interaction | 0.06  6.78  8.77 | .815  .013  .020 | .002  .145  .180 | bvFTD first  vs. HC first | 0.042 | -0.910 |
|  |  |  |  |  |  | bvFTD third  vs. bvFTD first | 0.999 | 0.093 |
|  |  |  |  |  |  | HC third  vs. HC first | 0.008 | -2.239 |
|  |  |  |  |  |  | bvFTD third  vs. HC third | 0.042 | 0.915 |
| # Pairwise comparisons are shown when the interaction effect is significant. All *p*-values are FDR corrected. AD: Alzheimer’s disease; bvFTD: behavioral variant frontotemporal dementia; HCs: healthy controls. | | | | | | | | |

**Table S5.** Word properties: Full statistical results.

|  | **Groups** | ***t*** | ***p*-value** | **Cohen’s *d*** |
| --- | --- | --- | --- | --- |
| **Frequency** | AD patients  vs. HCs | 3.682 | .012 | 1.136 |
|  | bvFTD patients  vs. HCs | -3.177 | .018 | -0.980 |
| **Phonological neighborhood** | AD patients  vs. HCs | 0.236 | .815 | 0.073 |
|  | bvFTD patients  vs. HCs | 0.736 | .699 | 0.227 |
| **Length** | AD patients  vs. HCs | -0.515 | .813 | -0.159 |
|  | bvFTD patients  vs. HCs | -0.749 | .699 | -0.231 |
| **Semantic variability** | AD patients  vs. HCs | 0.279 | .815 | 0.086 |
|  | bvFTD patients  vs. HCs | 0.918 | .699 | 0.283 |
| All *p*-values are FDR corrected. AD: Alzheimer’s disease; bvFTD: behavioral variant frontotemporal dementia; HCs: healthy controls. | | | | |
