# Supplementary material for: Automated free speech analysis reveals distinct markers of Alzheimer’s and frontotemporal dementia
Source: PLoS One. 2024 Jun 6;19(6):e0304272. doi: 10.1371/journal.pone.0304272 (PMC11156374; doi:10.1371/journal.pone.0304272)
Supplement: S4 File — S6 Table. Main study: Additional performance metrics for the classification analyses. S7 Table. Generalizability tests: Additional performance metrics for the classification analyses. (DOCX) [file pone.0304272.s004.docx]

***Supporting information 4. Full machine learning results***

**Automated free speech analysis reveals distinct markers of Alzheimer’s and frontotemporal dementia**

Pamela Lopes da Cunha,^1,2,¶^ Fabián Ruiz,^1,¶^ Franco Ferrante,^1,2,3^

Lucas Federico Sterpin,^1^ Agustín Ibáñez,^1,4,5^ Andrea Slachevsky,^6,7,8,9^

Diana Matallana,^10,11,12^ Ángela Martínez-R,^13^ Eugenia Hesse,^1,14^ Adolfo M. García,^1,4,5,15,*^

^1^ Cognitive Neuroscience Center, Universidad de San Andrés, Victoria, Buenos Aires, Argentina

^2^ Consejo Nacional de Investigaciones Científicas y Técnicas (CONICET), Ciudad Autónoma de Buenos Aires, Argentina

^3^ Facultad de Ingeniería, Universidad de Buenos Aires (FIUBA), Ciudad Autónoma de Buenos Aires, Argentina

^4^ Latin American Brain Health (BrainLat) Institute, Universidad Adolfo Ibáñez, Santiago, Peñalolén, Región Metropolitana, Chile

^5^ Global Brain Health Institute, University of California San Francisco, San Francisco, California, United States; and Trinity College Dublin, Dublin, Ireland

^6^ Neuropsychology and Clinical Neuroscience Laboratory (LANNEC), Physiopathology Program – Institute of Biomedical Sciences (ICBM), Neuroscience and East Neuroscience Departments, Faculty of Medicine, University of Chile, Santiago, Chile.

^7^ Geroscience Center for Brain Health and Metabolism (GERO), Providencia, Santiago, Chile

^8^ Memory and Neuropsychiatric Center (CMYN), Neurology Department, Hospital del Salvador and Faculty of Medicine, University of Chile, Providencia, Santiago, Chile

^9^ Servicio de Neurología, Departamento de Medicina, Clínica Alemana-Universidad del Desarrollo, Las Condes, Región Metropolitana, Chile

^10^ Instituto de Envejecimiento, Departamento de Psiquiatría (Programa PhD Neurociencias), Facultad de Medicina, Pontificia Universidad Javeriana, Bogotá, Colombia

^11^ Centro de Memoria y Cognición, Intellectus, Hospital Universitario San Ignacio Bogotá, San Ignacio, Colombia

^12^ Departamento de Salud Mental, Hospital Universitario Santa Fe de Bogotá, Bogotá, Colombia

^13^ Escuela de Medicina y Ciencias de la Salud, Universidad del Rosario, Bogotá, Colombia

^14^ Departamento de Matemática, Universidad de San Andres, Victoria, Buenos Aires, Argentina

^15^ Departamento de Lingüística y Literatura, Facultad de Humanidades, Universidad de Santiago de Chile, Estación Central, Santiago, Chile

***Corresponding author:**

E-mail: [adolfo.garcia@gbhi.org](mailto:adolfo.garcia@gbhi.org)

**Table S6.** Main study: Additional performance metrics for the classification analyses.

| **Group** | **AUC** | **Accuracy** | **Precision** | **Recall** | **F1 score** | **UAR** |
| --- | --- | --- | --- | --- | --- | --- |
| AD patients  vs. HCs | 0.71 (0.14) | 0.71 (0.14) | 0.73 (0.18) | 0.71 (0.20) | 0.70 (0.15) | 0.71 (0.14) |
| bvFTD patients vs. HCs | 0.71 (0.14) | 0.71 (0.14) | 0.70 (0.16) | 0.80 (0.20) | 0.73 (0.14) | 0.71 (0.14) |
| HCs: healthy controls; AD: Alzheimer’s disease; bvFTD: behavioral variant frontotemporal dementia. | | | | | | |

**Table S7.** Generalizability tests: Additional performance metrics for the classification analyses.

| **Group** | **AUC** | **Accuracy** | **Precision** | **Recall** | **F1 score** | **UAR** |
| --- | --- | --- | --- | --- | --- | --- |
| AD patients  vs. HCs | 0.76 | 0.63 | 0.69 | 0.64 | 0.60 | 0.64 |
| bvFTD patients vs. HCs | 0.83 | 0.77 | 0.77 | 0.77 | 0.77 | 0.77 |
| HCs: healthy controls; AD: Alzheimer’s disease; bvFTD: behavioral variant frontotemporal dementia. | | | | | | |
